# *Semenovia gyirongensis* (Apiaceae), a new species from Xizang, China

**DOI:** 10.3897/phytokeys.82.13010

**Published:** 2017-06-29

**Authors:** Qun Ying Xiao, Jin Bo Tan, Hao Yu Hu, Song Dong Zhou, Xing Jin He

**Affiliations:** 1 Key laboratory of Bio-Resources and Eco-Environment of Ministry of Education, College of Life Science, Sichuan University, 610065 Chengdu, Sichuan, People’s Republic of China

**Keywords:** Apiaceae, new species, pollen, Qinghai-Tibetan Plateau, *Semenovia*, taxonomy

## Abstract

Based on morphology and molecular data, a new species *Semenovia gyirongensis* Q.Y.Xiao & X.J.He, from Gyirong County, Xizang, China, is described and illustrated. It is morphologically most similar to *S. malcolmii* (Hemsley & Pearson) Pimenov, but differs in its cylindric much-branched root, intensively branching long underground caudex with distinct nodes, narrowly ovate to ovate terminal leaf lobes, oblong bracts with obtuse-rounded or cuneate apex.

## Introduction

*Semenovia* Regel & Herder (Apiaceae, tribe ), an endemic Asiatic genus, occurs in southwest, central and east Asia, with its center of diversity in the Pamir mountains (Shen 1992; Pimenov and Leonov 1993; Pu and Watson 2005; Ukrainskaja 2015). Most species of *Semenovia* are narrow endemics and grow mainly in the mid-elevation to highland areas of mountains (Ukrainskaja et al. 2013; Ukrainskaja 2015). The latest revision of *Semenovia* was conducted by Ukrainskaja et al. (2013), who recognized 29 species. There are 6 species of *Semenovia* in China, two of which are endemic to Qinghai-Tibetan Plateau (QTP) (Ukrainskaja et al. 2013). *Semenovia* is a perennial herb with pinnate leaves, entire or branched caudex, unequal (outer ones are larger) or subequal outer and inner petals, small bracts and bracteoles, well developed styles, thinly and narrowly winged marginal ribs, filiform vittae, solitary vittae per vallecular and two (rarely four) on commissural surface (Regel and Herder 1866; Mandenova 1959; Alava 1987; Pu and Watson 2005). Caudex states (underground, overground or emergent; unbranched or branched; short or long branches) are regarded as the most important diagnostic characters within the genus *Semenovia* (Ukrainskaja et al. 2013; Ukrainskaja 2015).

According to morphological data, the genus *Semenovia* clearly differs from closely related genera *Tordyliopsis* DC. (well-developed, broad leafy bracts and bracteoles), *Zosima* Hoffm. (strongly inflated and broadly winged marginal ribs, dorsal vittae occupying furrows completely), *Kandaharia* Alava (very short styles, up to 0.5 mm long, strongly inflated and broadly winged marginal ribs, numerous commissural vittae), and *Pastinacopsis* Golosk (vallecular and commissural vittae obsolete) (Mandenova 1959; Alava 1987; Pimenov et al. 2000; Menemen and Jury 2001; Pu and Watson 2005; Ukrainskaja et al. 2013).

During examining specimens of *Semenovia*, we encountered one collection (*Z. Y. Wu et al*. *75-0676*, stored in HNWP, KUN and PE), which was collected from Gyirong County, Xizang, China and was unable to identify as any described species. In August 2016, we carried out field investigation to the exact locality and gathered both flowering and fruiting plant from the natural population. After thoroughly consulting relevant literatures (e.g. Mandenova 1959; Alava 1987; Vinogradova and Kamelin 1986; Ukrainskaja et al. 2013; Ukrainskaja 2015) and herbarium specimens, as well as comparing this taxon with all described species within the genus, we come to the conclusion that the specimens from Gyirong represent a hitherto undescribed species. Herein a new name *Semenovia gyirongensis* is proposed, and detailed descriptions and comments of this new species, as well as comparisons with its morphologically similar species are given.

## Material and methods

### Specimen examinations, field investigations and morphology observations

Related specimens deposited in C, CDBI, HNWP, K, KUN, NAS, PE, SZ, XJA and XJBI were studied. Protologues and images of type specimens were gathered fromTropicos (http://www.tropicos.org), JSTOR Global Plants (http://plants.jstor.org) and the International Plant Names Index (http://www. ipni.org). Herbarium acronyms followed Thiers (2016).

Sampling was conducted from type localities of *S. gyirongensis* (Gyirong County, Xizang) and *S. malcolmii* (Shuanghu, Nyima County, Xizang) during 2015–2016. Photographs in the field were made using a Nikon D7100 camera. The measurements of the morphological features were conducted using a vernier caliper. Mericarps were photographed using stereomicroscope Nikon SMZ 25 (Japan). Fruits from formaldehyde-acetic acid-alcohol (FAA) preserved material were used in the anatomical study. Pollen was examined from anthers collected directly in the field. The pollen grains were mounted on clean aluminum stubs using conducting carbon adhesive tabs, coated and then scanned with a JSM-7500F scanning electron microscope (SEM). General terminologies for this study followed Kljuykov et al. (2004). Voucher specimens were deposited in the herbarium of Natural History Museum of Sichuan University (SZ).

### DNA extraction, amplification and sequencing

Total genomic DNA was extracted from silica gel-dried leaves and herbarium materials according to the protocols of plant genomic DNA kit (Tiangen Biotech, Beijing, China). The internal transcribed spacer (ITS) and external transcribed spacer (ETS) of nuclear ribosomal DNA (nrDNA) were used for phylogenetic inference based on the previous study (Logacheva et al. 2010). The primer pairs ITS4 / ITS5 (White et al. 1990) and 18S-ETS (Baldwin and Markos 1998) / Umb-ETS (Logacheva et al. 2010) were used to amplify the ITS and ETS regions, respectively. Amplification was carried out in a 30µL volume with 2 µL plant total DNA, 10 µL ddH_2_O, 1.5 µL forward primer, 1.5 µL reverse primer and 15 µL 2 × Taq MasterMix (cwbio, Beijing, China). PCR cycling profile included a denaturing step at 95 °C for 4 min, followed by 35 cycles of 45 s at 95 °C, annealing at 54 °C for 45 s and extension at 72 °C for 1 min, with a final extension for 10 min at 72°C. Sequencing (both directions) was carried out using the amplification primers on an ABI 3730 sequencer at the Beijing Genomics Institute (BGI) in Beijing, China. All newly reported sequences were deposited in GenBank and accession numbers along with sample codes and localities were given in Suppl. material 1: Table S1.

### Sequence alignment and phylogenetic analysis

62 accessions were obtained from GenBank for the nrDNA ITS and ETS, and 4 were newly sequenced for this study (Suppl. material 1: Table S1), representing 56 species from 17 genera of tribe (plus the new species *S. gyirongensis*, a total of 22 species of *Semenovia* were included) and 2 species of *Conium*. Sequence data for the ITS 5.8S region were excluded from the analysis because they were unavailable for many previously published taxa. *Conium maculatum* L. and *Conium sphaerocarpum* Hilliard & Burtt were selected as outgroups (Ajani et al. 2008; Banasiak et al. 2013).

SeqMan (Burland 2000) was used to edit DNA sequences and obtain consensus sequences. DNA sequences were aligned with ClustalX ver. 2.1 (Larkin et al. 2007) and then adjusted manually using MEGA7 (Kumar et al. 2016). Topological incongruence the partition between ITS and ETS was tested using the incongruence length difference (ILD) test (Farris et al. 1994) implemented in PAUP* version 4.0b10 (Swofford 2003). The two markers were then combined and analyzed using Bayesian Inference (BI), Maximum Likelihood (ML), and Maximum Parsimony (MP). Pairwise nucleotide differences of unambiguously aligned positions were determined from the distance matrix option in PAUP* (Swofford 2003). The BI analysis was performed in MrBayes version 3.2 (Ronquist et al. 2012). MrModeltest version 2.2 (Nylander 2004) was used to select a best-model (GTR+G) of nucleotide substitution. Four simultaneous runs were performed using Markov chain Monte Carlo (MCMC) simulations for 20 million generations, starting from a random tree and sampling one tree every 1000 generations. The convergence and effective sample size (ESS) of each replicate were checked using Tracer v. 1.6.0 (Rambaut et al. 2013). The first 25% of obtained trees were discarded as burn-in and the remaining were used to calculate a 50% majority-rule consensus topology and posterior probability (PP) values. For the ML analysis, phylogenetic reconstruction was performed using RAxML-HPC BlackBox ver. 8.2.10 under the GTR+G nucleotide substitution model and 1000 rapid bootstraps on the CIPRES Science Gateway ver. 3.3 (Miller et al. 2010). The MP tree was obtained using the programs PAUP* version 4.0b10. Heuristic searches were replicated 1000 times with random taxon addition sequences, tree bisection-reconnection (TBR) branch swapping, and setting the maximum number of trees to 10,000. Bootstrap values were calculated from 1,000,000 replicate analyses using ‘fast’ stepwise-addition of taxa and only those values compatible with the majority-rule consensus tree were recorded.

## Result and discussion

### Morphological analysis

**Figure 1.**
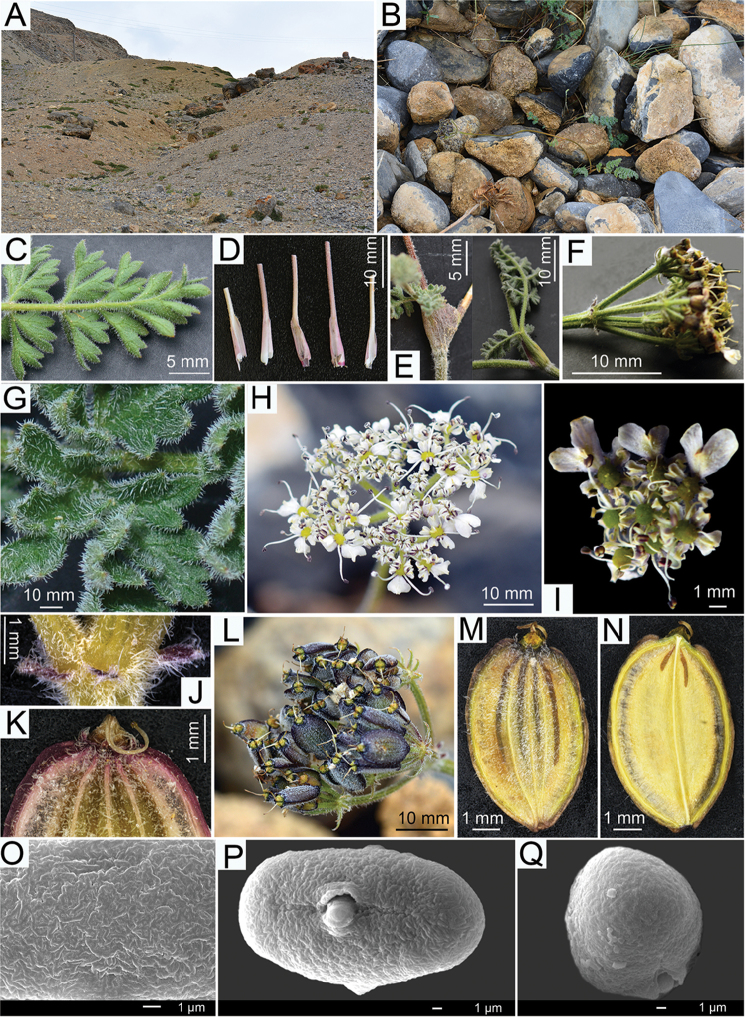
*Semenovia gyirongensis* **A–B** Habitat **C** Abaxial surface of primary pinnae **D** Basal leaf sheaths **E** Middle-upper cauline leaf sheaths **F** Rays **G** Adaxial surface of primary pinnae, showing hairs **H** Compound umbel **I** Umbellule **J** Bracts **K** Calyx teeth and stylopod **L** Infructescence **M** Dorsal side of mericarp **N** Commissural side of mericarp **O** Cerebroid ornamentation on equatorial plane of pollen grain **P** Tricolporate **Q** Rounded pollen polar ends.

**Figure 2.**
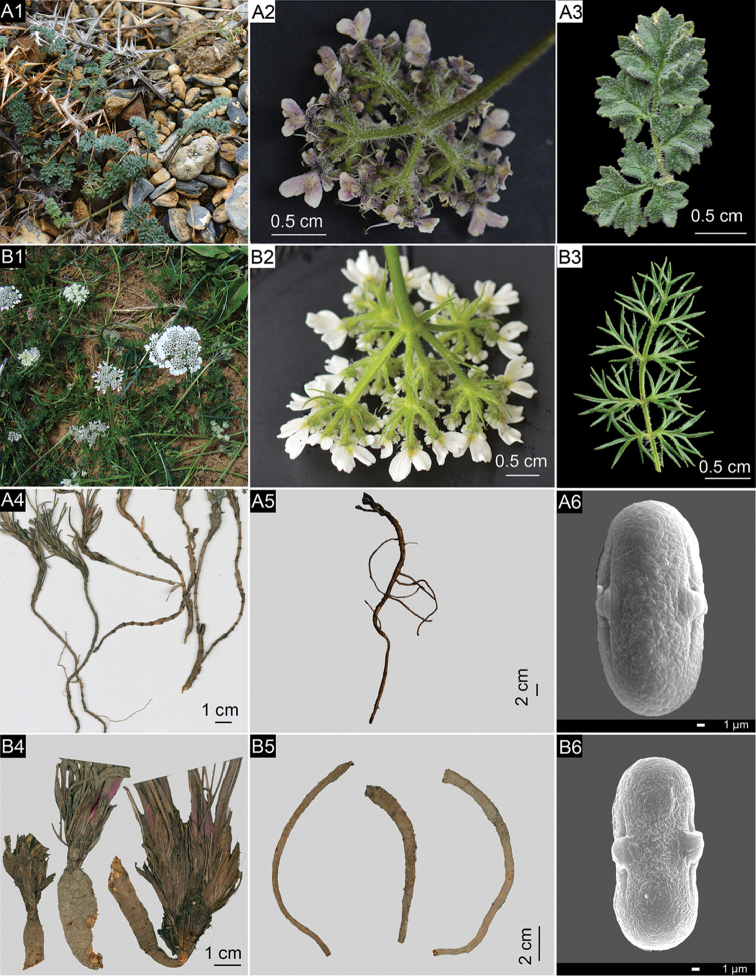
Diagnostic morphological characters of *Semenovia gyirongensis* (**A1–A6**) in comparison to the similar species *Semenovia malcolmii* (**B1–B6**) **A1–B1** Habitat **A2** Oblong minute bracts, apex obtuse-rounded or cuneate **B2** linear to narrowly ovate bracts, apex acute **A3** narrowly ovate to ovate ultimate leaf lobes **B3** linear to narrowly long-ovate ultimate leaf lobes **A4** Long-branched underground caudex with distinct nodes **B4** Short-branched overground or emergent caudex **A5** Cylindric and much-branched root **B5** Fusiform and unbranched root **A6** Ellipsoidal pollen grains **B6** Equatorially constricted pollen grains.

*S. gyirongensis* is a perennial polycarpic herb with very dense ribbon shaped hairs throughout, having cylindric much-branched roots, intensively branching long underground caudex with distinct nodes, covering rigid imbricate perished leaf sheaths and petioles, simple or sparingly branched stems, 2–3-pinnate leaves, narrowly ovate to ovate terminal leaf lobes, oblong bracts (minute), narrowly long-ovate bracteoles (2–4 mm), 5–10 rays, long styles (2.5–3 mm), mericarps elliptic or broadly elliptic, 4–7 mm long, thinly and narrowly winged marginal ribs, 4 dorsal vittae, and 2 commissural vittae (reaching 1/4 mericarp length) (Fig. 1, Fig. 2A1–A6). This description corresponds very well to the general characteristics of *Semenovia* (Regel and Herder 1866; Mandenova 1959; Alava 1987; Pu and Watson 2005), indicating that the new putative species under *Semenovia* is well justified.

These characters of *S. gyirongensis* allow for easy discrimination from morphologically similar species *S. malcolmii* (fusiform unbranched roots, unbranched to much-branched, short overground or emergent caudex, without distinct nodes, linear to narrowly long-ovate terminal leaf lobes and linear to narrowly ovate bracts, apex acute, Fig. 2B1–B6 and Table 1), *S. pamirica* (much dichotomously branched stems, 2–4 rays, and commissural vittae reaching 3/4 mericarp length, Suppl. material 1: Fig. S1D and Table 1), *S. vachanica* Ukrainskaja & Kljuykov (pinnatisect leaves, toothed terminal leaf lobes, linear-narrowly ovate bracts, apex almost filiform, Table 1) and the rest species of *Semenovia* not included in the phylogeny *S. pulvinata* Pimenov & Kljuykov (plants forming dense hemispheric cushion), *S. dissectifolia* Ukrainskaja & Kljuykov (soft fibrous remnant sheaths, vallecular vittae solitary to paired), *S. imbricata* Ukrainskaja & Kljuykov (alternately branched stems, commissural vittae almost reaching mericarp bases), *S. propinqua* (Aitch. & Hemsl.) Manden. (glabrous, much-branched stems, narrowly lanceolate bracts with membranous margins), *S. suffruticosa* (Freyn & Bornm.) Manden. (overground caudex, pinnate leaves, broadly triangular terminal leaf lobes), *S. heracleifolia* (Wolff) Hedge & Lamond (stems glabrous, basal leaves few, vittae reaching mericarp bases) and *S. macrocarpa* (Rech. f. & H. Riedl) Alava (2–3 rays, mericarps 8–10 mm long) (Alava 1987; Ukrainskaja et al. 2013).

**Table 1.** Main morphological difference between *Semenovia gyirongensis* and its morphological allies.

Charactes	*S. gyirongensis*	*S. malcolmii*	*S. pamirica*	*S. vachanica*
Hairs	very dense	sparse or dense	dense	dense
Root	cylindric, much-branched	fusiform, unbranched	incomplete material	incomplete material
Caudex	underground, much-branched, long branches, with distinct nodes	overground or emergent, unbranched to much-branched, short branches, without distinct nodes	overground or emertent, much-branched, short branches, without distinct nodes	underground, much-branched, long branches, without distinct nodes
Stem	simple or sparingly branched	simple or sparingly branched	much dichotomously branched	without branches or with a single branch above
Basal leaves	2–3-pinnate	2–3-pinnatisect	simple-pinnate	pinnatisect
Terminal leaf lobes	0.5–2 mm, narrowly ovate to ovate	0.7–5.7 mm, linear to narrowly long-ovate	2–4 mm, linear	0.5–2 mm, toothed
Rays	5–10, 1–2 cm	4–12, 0.5–3.5 cm	2–4, 1.5–2.5 cm	2 – 5, 1.5–2 cm
Bracts	minute, 0.4–1.5 mm oblong, apex obtuse-rounded or cuneate	2–8 mm, linear to narrowly ovate, apex acute	2–4 mm ovate-narrowly ovate, apex acute, margin white-scarious	4–6 mm, linear-narrowly ovate, apex almost filiform, white-margined
Commissural vittae	1/4 length of mericarp	1/4 length of mericarp to base	3/4 length of mericarp	short, not reaching fruit base

### Phylogenetic analysis

**Figure 3.**
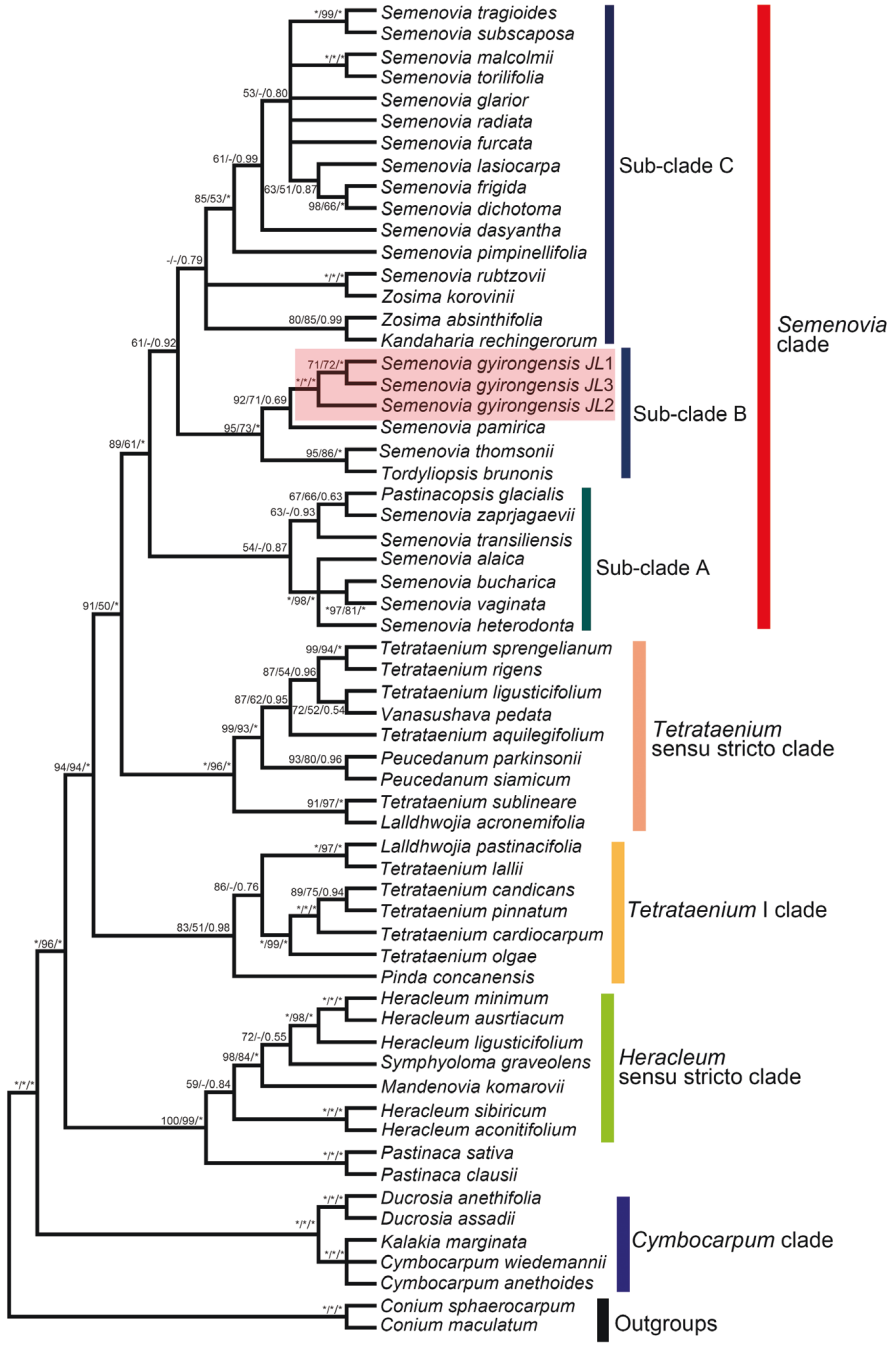
Phylogenetic tree inferred from tribe and outgroups of combined ITS/ETS dataset based on Bayesian inference (BI) method. The names of the major clades follow the study of Logacheva et al. (2010). Support values only those greater than 50% ML-BS, 50% MP-BS and 0.5 BI-PP are shown along the branches. Numbers on the branches indicated ML-BS/ MP-BS/ BI-PP, respectively. Asterisks denoted (*) the values of 100/100/1.00 for ML-BS/ MP-BS/ BI-PP. Dshes (-) indicated ML-BS and MP-BS values <50%.

The matrix of combined nrDNA ITS and ETS data had an aligned length of 775 positions, of which 310 were parsimony informative, 283 were constant, and 182 autapomorphic characters. The results of the ILD test for those 66 accessions common to both ITS and ETS datasets revealed that these loci yield significantly different phylogenetic estimates (P = 0.001). However, numerous reports indicated that the results of an ILD test do not adequately assess data combinability (e.g. Yoder et al. 2001; Barker and Lutzoni 2002; Hipp et al. 2004). Despite the incongruence of these data, the topologies of the ITS- and ETS-derived trees did not conflict. Meanwhile, the analysis of the combined dataset using ML, MP and BI yielded similar trees and had higher MP Bootstrap values (MP-BS), ML Bootstrap values (ML-BS) and BI posterior probabilities (BI-PP). The Bayesian majority rule consensus tree based on combined analysis was presented in Fig. 3. ML-BS, MP-BS and BI-PP values were showed along the branches.

Based on our reconstructed phylogeny, 5 major evolutionary clades (*Cymbocarpum* clade, *Heracleum* sensu stricto clade, *Semenovia* clade, *Tetrataenium* I clade and *Tetrataenium* sensu stricto clade) of tribe were identified (Fig. 3), which was consistent with previous works (Logacheva et al. 2010). The *Semenovia* clade was well supported (ML-BS 89%; MP-BS 61%; BI-PP 1.00) comprising *Zosima*, *Semenovia* and the monotypic genera *Tordyliopsis*, *Pastinacopsis* and *Kandaharia* and could be divided into three sub-clades (A, B, and C). The subclade B was strongly supported (ML-BS 95%; MP-BS 73%; BI-PP 1.00), but subclade A (ML-BS 54%; MP-BS <50%; BI-PP 0.87) and subclade C (ML-BS <50%; MP-BS <50%; BI-PP 0.79) were weakly supported (Fig. 3). The monotypic genera *Pastinacopsis* fell into sub-clade A with 6 species of *Semenovia*, while two species of *Zosima* and the monotypic genera *Kandaharia* intermixed within sub-clade C with the largest number of *Semenovia* taxa (12 species). Subclade B consisted of *Tordyliopsis brunonis* DC., *S. gyirongensis*, *S. pamirica* (Lipsky) Mandenova and *S. thomsonii* (C.B.Clarke) Mandenova (Fig. 3). Within sub-clade B, three accessions of *S. gyirongensis* formed a well monophyletic clade (MP-BS 100%; ML-BS 100%; BI-PP 1.00), as a sister group to *S. pamirica* (Fig. 3). The genus *Semenovia* is not monophyletic based on these phylogenies (and neither is *Zosima*) (Fig. 3). The circumscription of all genera within the *Semenovia* clade should be revised, but this is out of the scope of the present study.

In the concatenated data sets, pairwise sequence divergence estimates for the examined taxa of the *Semenovia* clade ranged from 0.00% (between *S. gyirongensis* JL1 and JL3) to 8.01% (between *Semenovia vaginata* Pimenov and *Zosima absinthifolia* Link) with a mean value 4.4%. Sequence comparisons between the three accessions of *S. gyirongensis* resulted in low pairwise divergence values of 0% to 0.14%, but *S. gyirongensis* and its closely related species *S. malcolmii* (4.96–5.10%), *S. pamirica* (4.16–4.3%), *T. brunonis* (3.45–3.59%) and *S. thomsonii* (3.85–3.99%) yielded relatively high sequence divergence value (Suppl. material 1: Table S2), supporting the hypothesis that *S. gyirongensis* is a distinct taxon.

### Geographical distribution

Geographically, *S. gyirongensis* is close or adjacent to *T. brunonis*, *S. pamirica*, *S. malcolmii* and *S. thomsonii* but do not overlap (Suppl. material 1: Fig. S2). *S. gyirongensis* is only known from the type locality, Gyirong County, Xizang, China. *T. brunonis* is distributed in Bhutan, Nepal, India (Sikkim, Himachal Pradesh, Uttarakhand) and also in South Xizang, but grows in subalpine moist dwarf scrubs, among shrubs and boulders (Pu and Watson 2005; Kumar et al. 2014). *S. pamirica* is confined to Pamiro-Alai and Central Asia (Shishkin 1968). *S. malcolmii* occurs in the QTP and adjacent regions, but never in Gyirong County. *S. thomsonii* is in Jammu, Kashmir and in whole India (Ukrainskaja et al. 2013) (Suppl. material 1: Fig. S2).

### Conclusion

Taking the morphology, molecular and geographical distribution evidences into consideration, it is thus clear that *S. gyirongensis* should be recognized as a new, distinct species of *Semenovia*.

### Taxonomic treatment

#### Semenovia gyirongensis

Taxon classificationPlantaeApialesApiaceae

Q.Y.Xiao & X.J.He sp. nov.

urn:lsid:ipni.org:names:77163815-1

Figure 1 , 2A1–A6 , 4 , Suppl. material 1: Fig. S1A–B, S3

##### Type.

**China**: Xizang, Gyirong County, Woma village, near Longda, 28°45.01'N, 85°18.22'E, 4023 m, 30 July 2016, *xqy*-*20160730*-*01* (holotype SZ; isotypes SZ).

##### Diagnosis.

*Semenovia gyirongensis* is most similar to *S. malcolmii*, but can be easily distinguished by its roots (cylindric much-branched vs. fusiform unbranched), caudex (intensively branching, long, underground, with distinct nodes vs. unbranched to much-branched, short, overground or emergent, without distinct nodes), terminal leaf lobes (narrowly ovate to ovate vs. linear to narrowly long-ovate), and bracts (oblong, apex obtuse-rounded or cuneate vs. linear to narrowly ovate, apex acute).

It is also similar to the closely related species *S. pamirica*, but differs in stems (simple or sparingly branched vs. much dichotomously branched), rays (5–10 vs. 2–4), and commissural vittae length (reaching 1/4 mericarp length vs. reaching 3/4 mericarp length).

**Figure 4.**
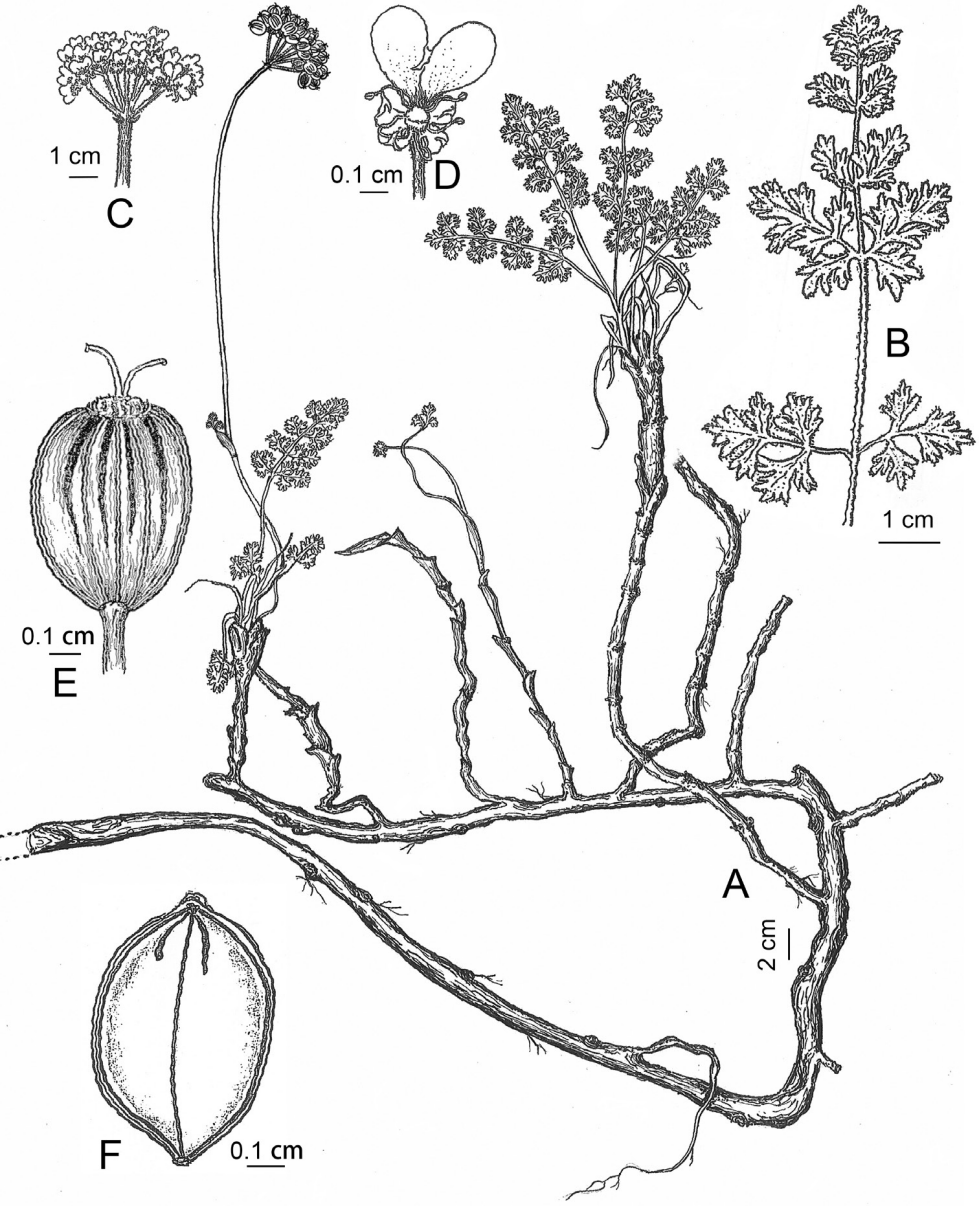
Illustrations of *Semenovia gyirongensis* (from the holotype) **A** Habit **B** Basal leaf **C** Compound umbel **D** Flower **E** Dorsal surface of mericarp **F** Commissural surface of mericarp.

##### Description.

Herbs perennial, polycarpic, grayish-green, 8–38 cm high, very dense ribbon shaped hairs throughout, with intensively branching long (up to 40 cm) underground caudex having distinct nodes (rooting at the nodes). Root long, cylindric, much-branched. Stems simple or sparingly branched, rigid, at the base covered with straw-yellow rigid imbricate perished leaf sheaths and petioles. Basal leaves rosulate, 5–21 cm long, numerous, very dense hairs on both sides; petioles (3–15 cm) longer than or nearly equal to blades; sheaths narrowly long-ovate, lavender; blades linear or narrowly-ovate in outline, 2–3-pinnate, primary pinnae 5–8 pairs; terminal leaf lobes (0.5–2 mm, narrowly ovate to ovate). Low cauline leaves similar to basal leaves but smaller, with narrowly long-ovate sheaths; middle-upper cauline leaves gradually reduced, sessile, with soft ovate sheaths. Compound umbels with 4–10 rays (1–2 cm, sub-equal length), slightly thickened in fruit. Central umbels broader than lateral umbels, up to 3.5 cm in diameter, compact. Bracts 2–5 (minute, 0.4–1.5 mm), oblong, apex obtuse-rounded or cuneate, caducous. Umbellets 0.8–1.8 cm in diameter in fruit, 6–15 flowered; bracteoles 4–6, purplish green, narrowly long-ovate, 2–4 mm; calyx teeth small, narrowly ovate. Petals broadly obovate or narrowly ovate, adaxially whitish-yellow, abaxially purplish-yellow, puberulent on both sides, outer flowers of the umbel radiant with outer petals enlarged, unequally emaginate at the tip, with narrow lobule bent inwards. Stylopods short-conic, wavy at the margin, yellow-green, 0.3–0.5 × 0.65–0.9 mm; styles reflexed, 2–3 mm long. Fruits with slender carpophore, bifurcate to the base; mericarps strongly dorsally compressed, elliptic or broadly elliptic in outline, 4–7 × 2–5 mm, on dorsal surface densely covered by thin hairs. Dorsal ribs filiform and marginal ribs narrowly-winged (0.2–0.5 mm broad). Vittae filiform, 4 on dorsal surface (1/2–3/4 length mericarp), 2 on commissure surface (short, about 1/4 as long as mericarp).

**Fruit anatomy.** Exocarp is formed by one layer of small cells. Outer mesocarp layer is of thin-walled parenchyma cells; inner mesocarp (hypendocarp) is consisted of thick-walled lignified fibrous cells. Five ridges are found on each mericarp. Vascular bundles are thin in dorsal ridges, broad in marginal ridges and commissural side. There are 4 dorsally and 2 ventrally vittae. Endoderm is located as one line under the vittae and seems to be integrated with the spermoderm. The seed is composed of endosperm and spermoderm with a thickened cell wall (Suppl. material 1: Fig. S3).

**Pollen morphology**. The pollen grains are isopolar symmetric, the aperture is tricolporate type. The pollen shape is prolate with an ellipsoidal equatorial outline, the polar ends are rounded and the ornamentation is cerebroid. Polar axis (P) = 26.53 ± 0.85 µm, equatorial axils (E) = 13.43 ± 0.9 µm (n = 20) (Fig. 1O–Q and Fig. 2A6).

**Phenology.** The species was found flowering in July–September, fruiting in August–October.

##### Distribution and habitat.

*S. gyirongensis* is only known from the type locality, China, Xizang, Gyirong County, Woma village, near Longda (Suppl. material 1: Fig. S2). It grows on screes, rocky slopes and sandy places, at elevations between 4000 and 4150 m.

##### Etymology.

The specific epithet is derived from the type locality, Gyirong County in Xizang, China.

**Conservation status.** *S. gyirongensis* is hitherto known only from Gyirong County (the type locality) where it usually grows on screes, rocky slopes and sandy places, locally common. In field investigation, we found that the area is subjected to overgrazing pressure and only a handful of individuals can escape from eating or trampling, ultimately blossoming and fruiting. Because of its localized distribution and grazing pressure, it should be assessed as “Vulnerable” (VU) according to the IUCN (2016).

##### Additional specimens examined

**(paratypes). China**: Xizang, Gyirong County, near Longda, 5 July 1975, *Z. Y. Wu et al*. *75-0676* (barcode: *KUN0565801*!, *PE 00756653*!, *PE 00756650*! and *HNWP 53717*!).

## Supplementary Material

XML Treatment for Semenovia gyirongensis

## Supplementary materials

Supplementary material 1 Supporting Information Data type: species data Explanation note: The following Supporting Information is available for this article: Figure S1–S3 and Tables S1–S2. This dataset is made available under the Open Database License (http://opendatacommons.org/licenses/odbl/1.0/). The Open Database License (ODbL) is a license agreement intended to allow users to freely share, modify, and use this Dataset while maintaining this same freedom for others, provided that the original source and author(s) are credited.Qun Ying Xiao, Jin Bo Tan, Hao Yu Hu, Song Dong Zhou, Xing Jin He
