# Supplementary material for: Semenovia gyirongensis (Apiaceae), a new species from Xizang, China
Source: PhytoKeys. 2017 Jun 29;(82):57–72. doi: 10.3897/phytokeys.82.13010 (PMC5546390; doi:10.3897/phytokeys.82.13010)
Supplement: Supplementary material 1 — Supporting Information [file phytokeys-82-057-s001.doc]

***Phytokeys* Supporting Information**

Article title: ***Semenovia gyirongensis* (Apiaceae), a new species from Xizang, China**

Authors: Qunying Xiao, Jinbo Tan, Haoyu Hu, Songdong Zhou & Xingjin He^*^

*Key laboratory of Bio-Resources and Eco-Environment of Ministry of Education*, *College of Life Science*, *Sichuan University*, *610065 Chengdu*, *Sichuan*, *People*’*s Republic of China* (**corresponding author*’*s email*: [*xjhe@scu*.*edu.cn*](mailto:xjhe@scu.edu.cn))

The following Supporting Information is available for this article: Figure S1–S3 and Tables S1–S2.

**
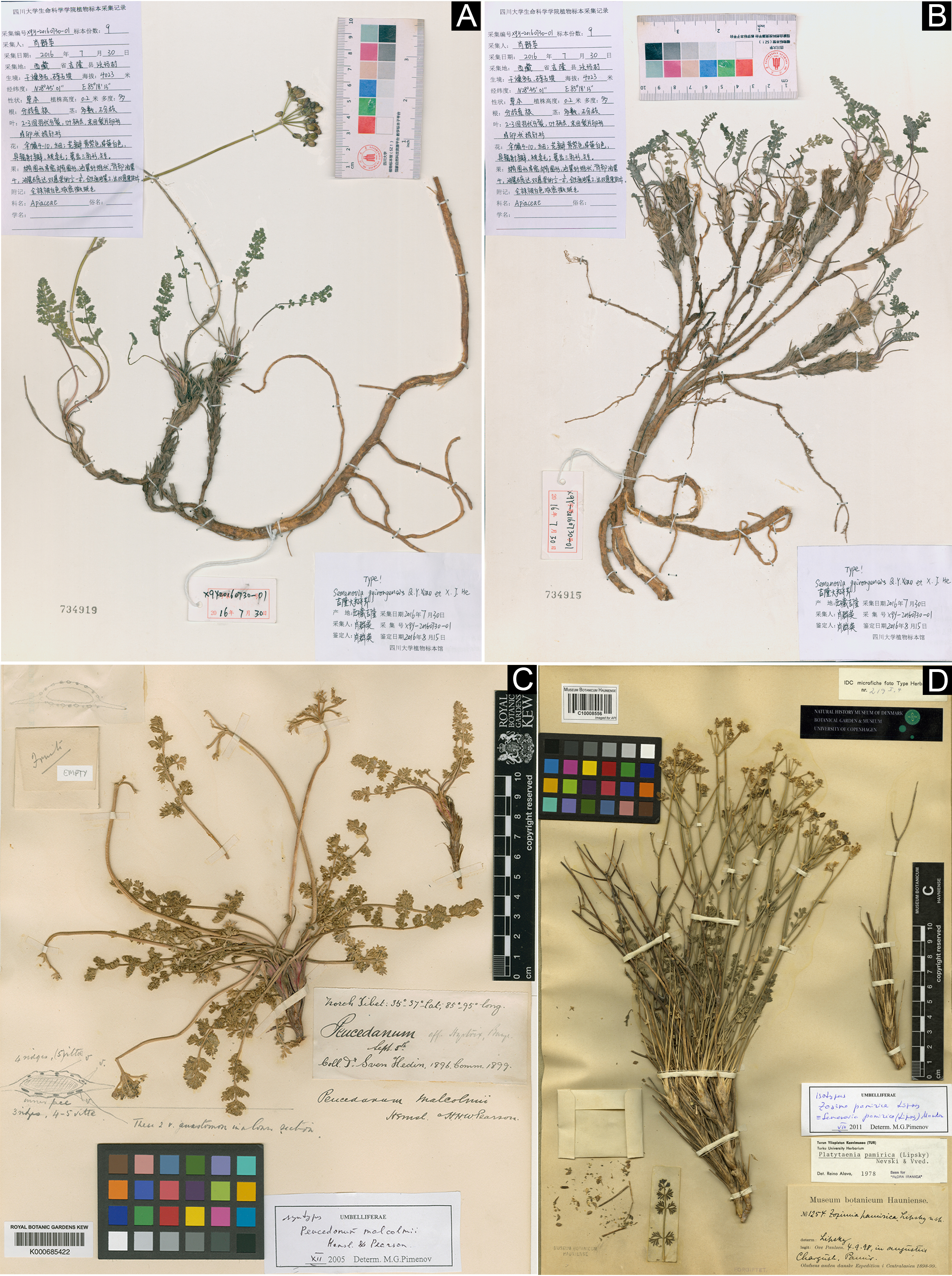
**

**Figure S1.** Types of *Semenovia* *gyirongensis, S. malcolmii* and *S. pamirica.* **A**–B Isotypes of *Semenovia* *gyirongensis* **C** Lectotype of *S*. *malcolmii* designated by Pimenov **D** Isotype of *S. pamirica*

*
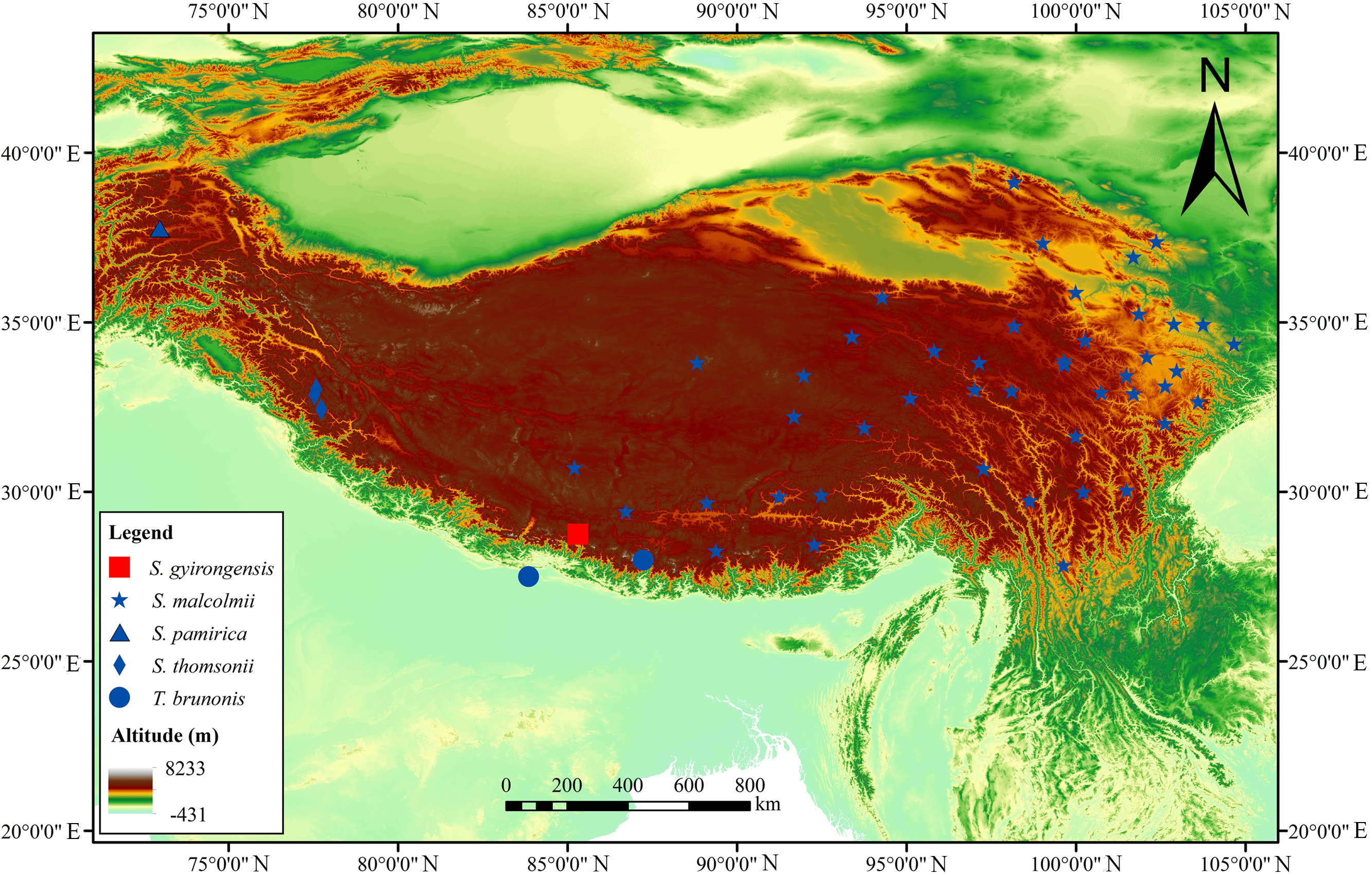
*

**Figure S2**. The known geographical distribution of *Semenovia gyirongensis* (red squares), *S. malcolmii* (blue pentagons), *S. pamirica* (blue triangles), *S. thomsonii* (blue lozenges) and *Tordyliopsis brunonis* (blue circles).


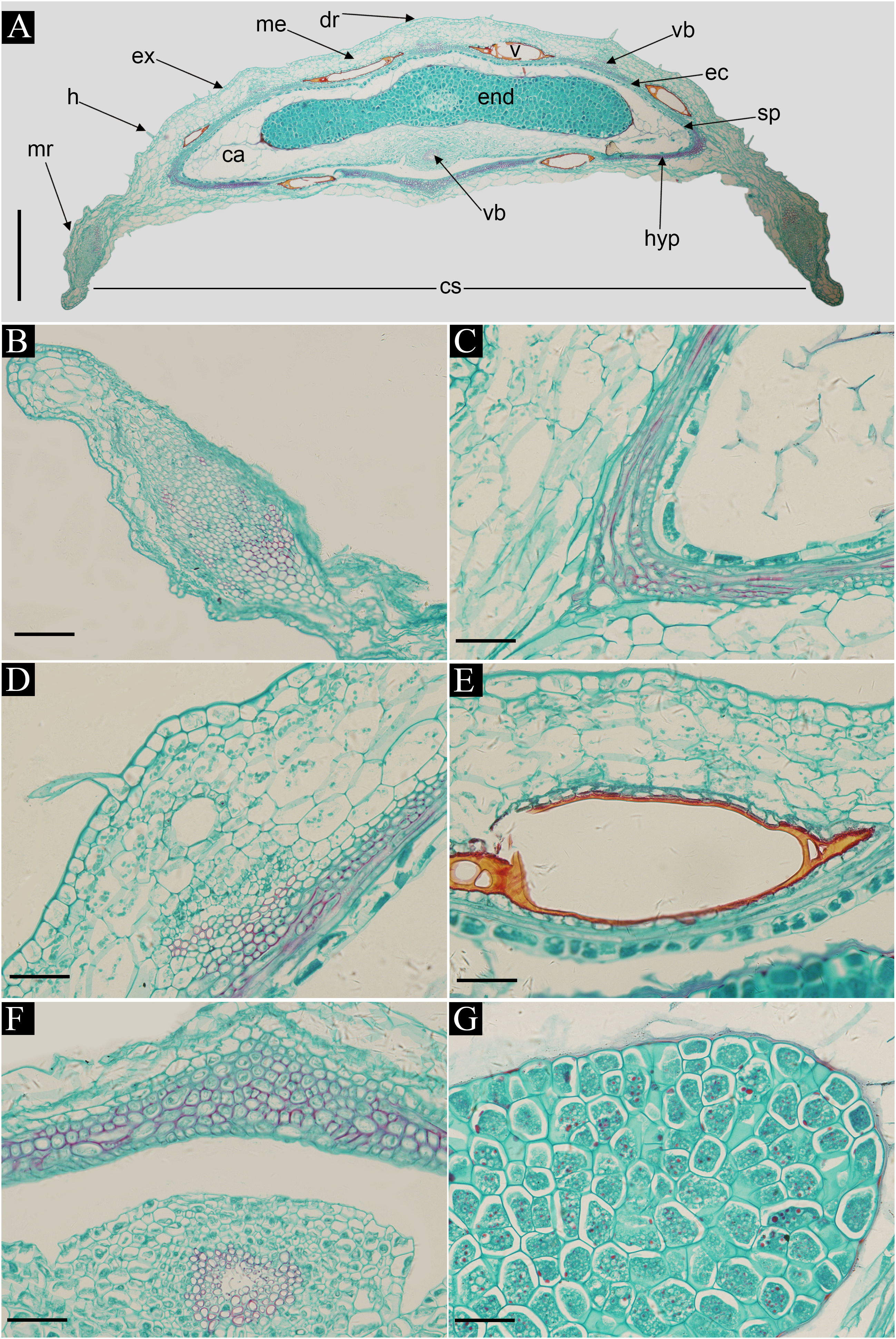


**Figure S3.** Transverse section of mericarp of *Semenovia gyirongensis* **A** Transverse section of mericarp **B** Marginal rib **C** Seed cavity **D** Dorsal rib **E** Dorsal vittae **F** Vascular bundles in commissural side **G** Endosperm. Scale bars: A = 500 µm; B = 100 µm; C, D, E, F and G= 50 µm (Legend: ca: seed cavity, cs: commissural side, v: vittae, ec: endocarp, end: endosperm, ex: exocarp, dr: dorsal rib, h: hair, hyp: hypendocarp (mesocarp fibres), me: outer mesocarp, mr: marginal rib, sp: spermoderm, vb: vascular bundle).

***Table S1****. Previously published nrDNA ITS (= ITS1, 5.8S rRNA gene and ITS2) and ETS accessions of tribe Tordylieae (Apiaceae) obtained from GenBank, with these sequences from the study of Logacheva et al. 2010. Taxa, sequences and GenBank accession numbers are provided. For each of the 8 newly obtained sequences, voucher information, DNA accession, and GenBank reference numbers are given.*

| Taxa | Sequence | Accession number |
| --- | --- | --- |
| *Conium maculatum* L. | ITS, ETS | GU266024, GU266175 |
| *Conium sphaerocarpum* Hilliard & Burtt | ITS, ETS | GU265958, GU266103 |
| *Cymbocarpum wiedemannii* Boiss. | ITS, ETS | GU291352, GU291351 |
| *Cymbocarpum anethoides* DC. ex C.A.Mey. | ITS, ETS | GU190156, GU291350 |
| *Ducrosia anethifolia* (DC.) Boiss. | ITS1, ITS2, ETS | AY941268, AY941296, FJ807503 |
| *Ducrosia assadii* Alava | ITS, ETS | DQ427043, GU190152 |
| *Heracleum aconitifolium* Woronow | ITS1, ITS2, ETS | AF008625, AF009104, FJ807504 |
| *Heracleum sibiricum* L. | ITS, ETS | EF043030, FJ807513 |
| *Heracleum austriacum* L. subsp. *siifolium* (Scop.) Nyman | ITS, ETS | EU594891, GU190147 |
| *Heracleum ligusticifolium* M.Bieb. | ITS1, ITS2, ETS | DQ996588, DQ996589, FJ807508 |
| *Heracleum minimum* Lam. | ITS, ETS | DQ996572, FJ807510 |
| *Kandaharia rechingerorum* Alava | ITS, ETS | DQ427052, GU190150 |
| *Kalakia marginata* (Boiss.) Alava | ITS1, ITS2, ETS | FJ861172, FJ828989, FJ807514 |
| *Lalldhwojia acronemifolia* (H.Wolff) M.F.Watson ex D.G.Long | ITS, ETS | DQ427044, GU190148 |
| *Lalldhwojia pastinacifolia* Pimenov & Kljuykov | ITS1, ITS2, ETS | EU526117, EU526118, GU190146 |
| *Mandenovia komarovii* (Manden.) Alava | ITS, ETS | EF043009, FJ807515 |
| *Pastinaca clausii* (Ledeb.) Calest. | ITS, ETS | DQ996579, GU190149 |
| *Pastinaca sativa* L. | ITS1, ITS2, ETS | FJ861173, FJ829023, FJ807516 |
| *Pastinacopsis glacialis* Golosk. | ITS1, ITS2, ETS | FJ861174, FJ828990, FJ807517 |
| *Peucedanum parkinsonii* Fedde ex H.Wolff | ITS, ETS | GU190154, GU190155 |
| *Peucedanum siamicum* Craib | ITS, ETS | GU190153, GU291348, GU291349 |
| *Pinda concanensis* (Dalzell) P.K.Mukh. & Constance | ITS1, ITS2, ETS | FJ861175, FJ828991, FJ807518 |
| *Semenovia alaica* Lazkov | ITS1, ITS2, ETS | FJ861176, FJ828992, FJ807519 |
| *Semenovia bucharica* ( Schischk.) Manden. | ITS1, ITS2, ETS | FJ861177, FJ828993, FJ807520 |
| *Semenovia dasycarpa* (Regel & Schmalh.) Korovin ex Czer. | ITS1, ITS2, ETS | FJ861178, FJ828994, FJ807521 |
| *Semenovia dichotoma* (Boiss.) Manden. | ITS1, ITS2, ETS | AY941287, AY941315, FJ807522 |
| *Semenovia frigida* (Boiss. & Hausskn. ex Boiss.) Manden. | ITS, ETS | DQ427039, FJ807523 |
| *Semenovia furcata* Korovin | ITS1, ITS2, ETS | FJ861179, FJ828995, FJ807524 |
| *Semenovia heterodonta* (Korovin) Manden. | ITS1, ITS2, ETS | FJ861181, FJ828997, FJ807526 |
| *Semenovia lasiocarpa* (Boiss.) Manden. | ITS1, ITS2, ETS | FJ861182, FJ828998, FJ807527 |
| *Semenovia torilifolia* (Boissieu) Pimenov, Pimenov et al. 136 | ITS1, ITS2, ETS | FJ861183, FJ828999, FJ807528 |
| *Semenovia pamirica* (Lipsky) Manden. | ITS1, ITS2, ETS | FJ861184, FJ829000, FJ807529 |
| *Semenovia pimpinelloides* (Nevski) Manden. | ITS1, ITS2, ETS | FJ861185, FJ829001, FJ807530 |
| *Semenovia radiata* (Rech.f. & Riedl) Alava | ITS, ETS | DQ427040, FJ807531 |
| *Semenovia rubtzovii* (Schischk.) Manden. | ITS1, ITS2, ETS | FJ861186, FJ829002, FJ807532 |
| *Semenovia subscaposa* (Rech.f.) Alava | ITS1, ITS2, ETS | FJ861187, FJ829003, FJ807533 |
| *Semenovia thomsonii* (C.B.Clarke) Manden. | ITS1, ITS2, ETS | FJ861188, FJ829004, FJ807534 |
| *Semenovia thomsonii* (C.B.Clarke) Manden. subsp. *glabrior* C.B.Clarke | ITS1, ITS2, ETS | FJ861180, FJ828996, FJ807525 |
| *Semenovia tragioides* (Boiss.) Manden. | ITS1, ITS2, ETS | AY941288, AY941316, FJ807535 |
| *Semenovia transiliensis* Regel & Herder | ITS1, ITS2, ETS | EU526113, EU526114, FJ807536 |
| *Semenovia vaginata* Pimenov | ITS1, ITS2, ETS | FJ861190, FJ829006, FJ807537. |
| *Semenovia zaprjagaevii* Korovin | ITS1, ITS2, ETS | FJ861206, FJ829007, FJ807538 |
| *Symphyoloma graveolens* C.A.Mey. | ITS, ETS | EF043015, FJ807539 |
| *Tetrataenium aquilegifolium* (C.B.Clarke) Manden. | ITS1, ITS2, ETS | FJ861192, FJ829021, FJ807541 |
| *Tetrataenium candicans* (DC.) Manden. | ITS1, ITS2, ETS | FJ861196, FJ829011, FJ807545 |
| *Tetrataenium cardiocarpum* (Rech.f. & Riedl) Manden. | ITS1, ITS2, ETS | FJ861197, FJ829012, FJ807547 |
| *Tetrataenium lallii* (C.Norman) Cauwet et al. | ITS1, ITS2, ETS | FJ861198, FJ829013, FJ807548 |
| *Tetrataenium olgae* (Regel & Schmalh.) Manden. | ITS1, ITS2, ETS | FJ861199, FJ829014, FJ807549 |
| *Tetrataenium pinnatum* (C.B.Clarke) Manden. | ITS, ETS | DQ427034, FJ807550 |
| *Tetrataenium rigens* (DC.) Manden. | ITS1, ITS2, ETS | FJ861200, FJ829015, FJ807551 |
| *Tetrataenium sprengelianum* (Wight & Arn.) Manden. | ITS1, ITS2, ETS | FJ861195, FJ829010, FJ807544 |
| *Tetrataenium candolleanum* (Wight & Arn.) Manden. | ITS1, ITS2, ETS | FJ861193, FJ829009, FJ807542 |
| *Tetrataenium sublineare* (C.B.Clarke) Manden. ex Farille et al. | ITS1, ITS2, ETS | FJ861201, FJ829016, FJ807552 |
| *Tordyliopsis brunonis* DC. | ITS1, ITS2, ETS | FJ861202, FJ829017, FJ807553 |
| *Vanasushava pedata* (Wight) P.K.Mukh. & Constance | ITS1, ITS2, ETS | FJ861203, FJ829018, FJ807554 |
| *Zosima_absinthifolia* (Vent.) Link | ITS1, ITS2, ETS | FJ861204, FJ829019, FJ807555 |
| *Zosima korovinii* Pimenov | ITS1, ITS2, ETS | FJ861205, FJ829020, FJ807556 |
| *Semenovia malcolmii* (Hemsl. & H. Pearson) Pimenov., DNA No. SH1, China, Xizang Province, Nima County, Shuanghu, xqy2014091701 (SZ) | ITS, ETS | KX896238, KX896157 |
| *Semenovia gyirongensis* Q.Y.Xiao & X.J.He, sp. nov., DNA No. JL1, China, Xizang Province, Gyirong County, Woma village, near Longda, xqy-20160730-0101 (SZ) | ITS, ETS | KX896208, KX896127 |
| *Semenovia gyirongensis* Q.Y.Xiao & X.J.He, sp. nov., DNA No. JL2, Xizang Province, Gyirong County, near Longda, *Z*. *Y*. *WU et al*. *75-0676* (*HNWP* [*53717*](http://www.cvh.org.cn/spm/QTPMB/53717)) | ITS, ETS | KX896209, KX896128 |
| *Semenovia gyirongensis* Q.Y.Xiao & X.J.He, sp. nov., DNA No. JL3, China, Xizang Province, Gyirong County, Woma village, near Longda, xqy-20160730-01 (SZ) | ITS, ETS | KX896210, KX896129 |

**Table S2**. Pairwise sequence divergence in percentages of combined nrDNA ITS and ETS regions among representatives of the *Semenovia* clade.

|  | 1 | 2 | 3 | 4 | 5 | 6 | 7 | 8 | 9 | 10 | 11 | 12 | 13 | 14 | 15 | 16 | 17 | 18 | 19 | 20 | 21 | 22 | 23 | 24 | 25 | 26 | 27 | 28 |
| --- | --- | --- | --- | --- | --- | --- | --- | --- | --- | --- | --- | --- | --- | --- | --- | --- | --- | --- | --- | --- | --- | --- | --- | --- | --- | --- | --- | --- |
| 1 S. gyirongensis JL2 |  |  |  |  |  |  |  |  |  |  |  |  |  |  |  |  |  |  |  |  |  |  |  |  |  |  |  |  |
| 2 S. gyirongensis JL1 | 0.14 |  |  |  |  |  |  |  |  |  |  |  |  |  |  |  |  |  |  |  |  |  |  |  |  |  |  |  |
| 3 S. gyirongensis JL3 | 0.14 | 0.00 |  |  |  |  |  |  |  |  |  |  |  |  |  |  |  |  |  |  |  |  |  |  |  |  |  |  |
| 4 *T. brunonis* | 3.45 | 3.59 | 3.59 |  |  |  |  |  |  |  |  |  |  |  |  |  |  |  |  |  |  |  |  |  |  |  |  |  |
| 5 *S. pimpinellifolia* | 3.60 | 3.74 | 3.74 | 2.36 |  |  |  |  |  |  |  |  |  |  |  |  |  |  |  |  |  |  |  |  |  |  |  |  |
| 6 *S. thomsonii* | 3.85 | 3.99 | 3.99 | 1.79 | 2.49 |  |  |  |  |  |  |  |  |  |  |  |  |  |  |  |  |  |  |  |  |  |  |  |
| 7 *S. dasyantha* | 3.87 | 4.01 | 4.01 | 2.62 | 0.28 | 2.76 |  |  |  |  |  |  |  |  |  |  |  |  |  |  |  |  |  |  |  |  |  |  |
| 8 *S. frigida* | 3.99 | 4.13 | 4.13 | 3.03 | 0.97 | 3.17 | 0.97 |  |  |  |  |  |  |  |  |  |  |  |  |  |  |  |  |  |  |  |  |  |
| 9 *S. lasiocarpa* | 3.99 | 4.13 | 4.13 | 3.03 | 0.97 | 3.03 | 0.97 | 0.83 |  |  |  |  |  |  |  |  |  |  |  |  |  |  |  |  |  |  |  |  |
| 10 *S. dichotoma* | 4.16 | 4.30 | 4.30 | 3.19 | 1.11 | 3.33 | 1.11 | 0.14 | 0.97 |  |  |  |  |  |  |  |  |  |  |  |  |  |  |  |  |  |  |  |
| 11 *S. pamirica* | 4.16 | 4.30 | 4.30 | 3.61 | 3.48 | 3.88 | 3.75 | 4.16 | 3.88 | 4.32 |  |  |  |  |  |  |  |  |  |  |  |  |  |  |  |  |  |  |
| 12 *S. furcata* | 4.17 | 4.31 | 4.31 | 3.20 | 1.12 | 3.34 | 1.11 | 1.25 | 1.53 | 1.40 | 4.33 |  |  |  |  |  |  |  |  |  |  |  |  |  |  |  |  |  |
| 13 *S. tragioides* | 4.43 | 4.56 | 4.56 | 3.32 | 1.25 | 3.46 | 1.25 | 1.66 | 1.66 | 1.81 | 4.31 | 1.81 |  |  |  |  |  |  |  |  |  |  |  |  |  |  |  |  |
| 14 *P. glacialis* | 4.83 | 4.97 | 4.97 | 3.32 | 2.36 | 4.01 | 2.63 | 2.49 | 2.76 | 2.64 | 5.00 | 2.65 | 3.19 |  |  |  |  |  |  |  |  |  |  |  |  |  |  |  |
| 15 *S. malcolmii* | 4.96 | 5.10 | 5.10 | 4.00 | 2.49 | 4.27 | 2.49 | 2.89 | 2.62 | 2.91 | 4.85 | 3.06 | 3.18 | 4.14 |  |  |  |  |  |  |  |  |  |  |  |  |  |  |
| 16 *S. radiata* | 4.99 | 5.12 | 5.12 | 3.74 | 1.81 | 3.88 | 1.80 | 2.22 | 2.22 | 2.23 | 4.87 | 2.36 | 2.49 | 3.89 | 3.19 |  |  |  |  |  |  |  |  |  |  |  |  |  |
| 17 *S. rubtzovii* | 5.10 | 5.24 | 5.24 | 4.14 | 2.63 | 4.28 | 2.90 | 3.03 | 3.17 | 3.19 | 5.27 | 3.48 | 3.88 | 3.60 | 4.83 | 3.88 |  |  |  |  |  |  |  |  |  |  |  |  |
| 18 *S. glarior* | 5.10 | 5.23 | 5.23 | 3.86 | 1.94 | 4.27 | 1.93 | 2.48 | 2.20 | 2.50 | 4.99 | 2.64 | 2.77 | 4.01 | 3.72 | 3.05 | 4.41 |  |  |  |  |  |  |  |  |  |  |  |
| 19 *S. subscaposa* | 5.23 | 5.37 | 5.37 | 4.14 | 2.08 | 4.27 | 2.07 | 2.48 | 2.48 | 2.64 | 5.13 | 2.64 | 0.83 | 4.01 | 3.99 | 3.32 | 4.69 | 3.44 |  |  |  |  |  |  |  |  |  |  |
| 20 *S. torilifolia* | 5.25 | 5.39 | 5.39 | 4.29 | 2.78 | 4.42 | 2.77 | 3.18 | 2.90 | 3.20 | 5.13 | 3.35 | 3.33 | 4.43 | 0.28 | 3.47 | 5.12 | 4.01 | 4.14 |  |  |  |  |  |  |  |  |  |
| 21 *S. zaprjagaevii* | 5.52 | 5.66 | 5.66 | 4.15 | 3.61 | 4.83 | 3.74 | 3.45 | 3.73 | 3.62 | 5.70 | 4.46 | 4.16 | 2.90 | 5.66 | 5.14 | 4.84 | 5.25 | 4.97 | 5.96 |  |  |  |  |  |  |  |  |
| 22 *K. rechingerorum* | 5.52 | 5.66 | 5.66 | 4.70 | 3.05 | 4.83 | 3.18 | 3.86 | 3.59 | 4.03 | 5.83 | 4.04 | 4.16 | 4.43 | 4.97 | 4.72 | 4.42 | 4.55 | 4.83 | 5.26 | 5.39 |  |  |  |  |  |  |  |
| 23 *S. alaica* | 5.53 | 5.67 | 5.67 | 5.12 | 4.17 | 5.53 | 4.44 | 4.01 | 4.70 | 4.18 | 6.55 | 4.19 | 5.14 | 4.02 | 5.95 | 5.01 | 4.57 | 5.95 | 5.95 | 6.24 | 5.55 | 5.96 |  |  |  |  |  |  |
| 24 *S. transiliensis* | 5.70 | 5.84 | 5.84 | 4.87 | 3.76 | 5.15 | 3.89 | 3.76 | 4.03 | 3.91 | 5.87 | 4.21 | 4.60 | 3.49 | 5.84 | 5.17 | 5.01 | 5.29 | 5.42 | 6.14 | 5.02 | 5.57 | 5.17 |  |  |  |  |  |
| 25 *Z. korovinii* | 5.94 | 6.08 | 6.08 | 4.98 | 3.47 | 5.11 | 3.73 | 3.73 | 3.87 | 3.74 | 6.10 | 4.31 | 4.71 | 4.29 | 5.52 | 4.16 | 1.66 | 5.25 | 5.11 | 5.82 | 5.40 | 5.12 | 5.13 | 5.85 |  |  |  |  |
| 26 *S. bucharica* | 5.97 | 6.11 | 6.11 | 5.29 | 4.45 | 5.42 | 4.72 | 4.58 | 4.86 | 4.74 | 6.83 | 5.31 | 5.42 | 4.32 | 6.25 | 5.56 | 5.41 | 6.11 | 6.25 | 6.55 | 5.43 | 6.12 | 2.79 | 5.31 | 5.69 |  |  |  |
| 27 *S. heterodonta* | 5.96 | 6.10 | 6.10 | 5.42 | 4.60 | 5.83 | 4.86 | 4.72 | 5.13 | 4.87 | 6.69 | 5.43 | 5.55 | 4.73 | 6.38 | 5.83 | 5.55 | 6.38 | 6.38 | 6.68 | 5.98 | 6.11 | 3.06 | 5.73 | 6.38 | 3.34 |  |  |
| 28 *S. vaginata* | 6.34 | 6.48 | 6.48 | 5.94 | 5.27 | 6.07 | 5.53 | 5.38 | 5.52 | 5.56 | 6.80 | 6.13 | 6.23 | 5.12 | 6.48 | 6.24 | 6.22 | 6.48 | 7.03 | 6.78 | 6.50 | 6.90 | 3.60 | 6.55 | 7.05 | 2.64 | 3.89 |  |
| 29 *Z. absinthifolia* | 6.48 | 6.62 | 6.62 | 6.08 | 4.72 | 5.93 | 4.98 | 5.38 | 5.66 | 5.56 | 6.80 | 5.85 | 5.54 | 6.22 | 6.34 | 5.96 | 6.77 | 6.76 | 6.34 | 6.63 | 6.64 | 5.25 | 7.48 | 7.38 | 7.61 | 7.51 | 7.50 | 8.01 |
